# Modifications of Surgical Suction Tip Geometry for Flow Optimisation: Influence on Suction-Induced Noise Pollution

**DOI:** 10.1155/2018/3074819

**Published:** 2018-11-21

**Authors:** M. G. Friedrich, T. Tirilomis, J. M. Kollmeier, Y. Wang, G. G. Hanekop

**Affiliations:** ^1^Department of Thoracic, Cardiac and Vascular Surgery, University Medical Center Göttingen, Göttingen, Germany; ^2^Max Planck Institute for Biophysical Chemistry, Göttingen, Germany; ^3^Max Planck Institute for Dynamics and Self-Organization, Göttingen, Germany; ^4^Department of Anaesthesiology, University Medical Center Göttingen, Göttingen, Germany

## Abstract

**Introduction:**

Suction devices for clearing the surgical field are among the most commonly used tools of every surgeon because a better view of the surgical field is essential. Forced suction may produce disturbingly loud noise, which acts as a nonnegligible stressor. Especially, in emergency situations with heavy bleeding, this loud noise has been described as an impeding factor in the medical decision-making process. In addition, there are reports of inner ear damage in patients due to suction noises during operations in the head area. These problems have not been solved yet. The purpose of this study was to analyse flow-dependent suction noise effects of different surgical suction tips. Furthermore, we developed design improvements to these devices.

**Methods:**

We compared five different geometries of suction tips using an in vitro standardised setup. Two commercially available standard suction tips were compared to three adapted new devices regarding their flow-dependent (10–2000 mL/min) noise emission (dB, weighting filter (A), distance 10 cm) and acoustic quality of resulting noises (Hamilton fast Fourier analysis) during active suction at the liquid-air boundary. Noise maps at different flow rates were created for all five suction devices, and the proportion of extracted air was measured. The geometries of the three custom-made suction tips (new models 1, 2, and 3) were designed considering the insights after determining the key characteristics of the two standard suction models.

**Results:**

The geometry of a suction device tip has significant impact on its noise emission. For the standard models, the frequency spectrum at higher flow rates significantly changes to high-frequency noise patterns (>3 kHz). A number of small side holes designed to prevent tissue adhesion lead to increased levels of high-frequency noise. Due to modifications of the tip geometry in our new models, we are able to achieve a highly significant reduction of noise level at low flow rates (new model 2 vs. standard models *p* < 0.001) and also the acoustic quality improved. Additionally, we attain a highly significant reduction of secondary air intake (new model 2 vs. the other models *p* < 0.001).

**Conclusion:**

Improving flow-relevant features of the geometry of suction heads is a suitable way to reduce noise emissions. Optimized suction tips are significantly quieter. This may help us to reduce noise-induced hearing damage in patients as well as stress of medical staff during surgery and should lead to quieter operation theatres overall. Furthermore, the turbulence reduction and reduced secondary air intake during the suction process are expected to result in protective effects on the collected blood and thus could improve the quality of autologous blood retransfusions. We are on the way to evaluate potential benefits.

## 1. Background

Suction devices for clearing the surgical field are used by surgeons during almost every type of surgical procedure to obtain a better view of the surgical field. The suction device may also be used as a hook, for dissection, and removal of excess tissue. There are different types of suction devices for different types of surgical procedures. As disposables, they are cheap and effective, although not yet optimized regarding their noise emissions.

Tissue adhesion of the suction head to delicate tissues is a well-known problem, especially during forced suction, and can result in severe tissue damage [1, 2]. To prevent this, several rows of supplementary holes were introduced in commercially available suction tubes. The fact that this leads to massive noise increases, however, has been accepted up to now, although it is well known that forced suction at the liquid-air boundary results in a massive increase of noise emissions with levels up to 120 dB(A) [3–7]. Loud noise is a significant stressor in the operation theatre (OT) [4]. This is aggravated during emergencies with massive bleeding, where it is important to rapidly achieve a clear and dry surgical field to stop blood loss as fast as possible. In contradiction to that, it has been shown that quiet working environments help us to work more efficiently and reduce the rates of error [8].

Furthermore, loud suction noises near the inner ear may result in permanent hearing loss in patients undergoing surgery in the ear and temporal head area [1, 9, 10]. It is not evident why the most often used tool in the OT has not been optimized yet. This study therefore aims to demonstrate that even small modifications of the geometry of suction tips are able to significantly reduce harmful noise emissions.

## 2. Methods

The noise data of two commercially available standard suction heads (**A**: Hex Handle Adult Sump Sucker, NovoSci, Conroe, Texas, USA, **B**: Argyle Yankauer, Covidien, Mansfield, Massachusetts, USA) were analysed. Due to theoretical considerations, model 1 was changed on the basis of fluid dynamic modelling: rounded edges in the inflow, sharp edges in the discharge area, no flow deflections >45° like in standard model A. Based on data derived from an interim analysis and using the “trial and error” principle, three new devices were developed (Table 1).

**Figure 1:**
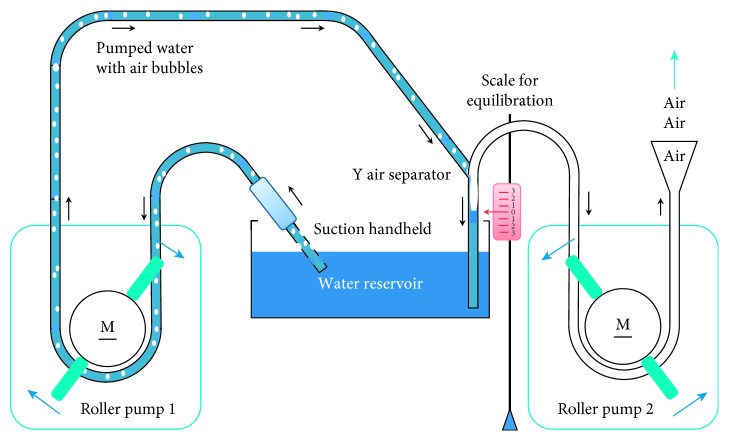
In vitro setup for noise mapping and measurement of aeration (mixing air in fluid), for a given liquid pumping rate (roller pump 1, rotation clockwise), the flow is increased by roller pump 2 (rotation counterclockwise) until the liquid column in the riser pipe is constant (equilibration). The pump rate of roller pump 2 corresponds to the proportion of pumped air. Adjustment of depth under water surface of the suction tip: set at maximum noise level at 1000 mL/min.

The devices were compared regarding to their flow-dependent (10–2000 mL/min) noise emission (dB(A) 10 cm) and acoustic quality (Fast Fourier Analysis, Hamming-window) during active suction at the liquid-air boundary (100, 200, 400,…, 2000 mL/min) using an in vitro setup (Figure 1) with a roller pump (Polystan, Type Modular No. 1603, Vaerlose, Denmark) and ¼-inch (6.3 mm) tube system (HMT-Medizintechnik GmbH, Maisach, Germany).

Noise emissions were measured at a 10 cm distance from the suction head and recorded in a standardised fashion over a period of 10 sec (electret condenser capsule microphone, ultralinear frequency range: 20 Hz-20 kHz, 32 Channel Mixer CM8000, Behringer Music Group, Germany, TASCAM DR-100 Digital Recorder, stored as uncompressed WAV-file in 96 kHz, 24 bit, edited in Hi-Res Editor software, TEAC Corporation, Tokyo, Japan).

Noise levels were also measured and recorded over 10-sec intervals (dB(A), Voltcraft sound level recorder SL-451, 125 ms peaks, 31.5 Hz-8 kHz, distance 10 cm). The frequency spectra were graphically displayed as a noise map (Fast Fourier Analysis as spectroscopy and spectrography during 5 sec, Hamming-filter, 20 Hz-20 kHz, FFT Size 8192, Sequoia 14.1.0.157 DC2, 64 bit, Magix Software GmbH, Berlin, Germany).

Flow-dependant secondary air intake was measured using a modified experimental setup, where the fluid was sucked up from a basin and returned via a large-bore Y-tube. At one arm of the Y-tube, another identical roller pump was used to separate the amount of secondary air from the liquid-air-mixture by keeping the level of the liquid column constant at ±1 cm (cf. Figure 1). The adjustment of the underwater diving depth of every suction tip was carried out by adjusting the maximum noise level at 1000 mL/min.

The experimental data from this setup were used to optimize suction tip geometry regarding noise emission. Necessary side holes should absorb less air at the liquid-air boundary. As shown in Table 1, three new models were developed. In new model 1, the angles of the supplementary holes are oriented towards the mainstream axis. The model 2 has the angles of the supplementary holes even more closely aligned to the mainstream axis. The geometry of the inlet area of the mainstream channel resembles that of a trumpet, to achieve continuous acceleration of flow. Additionally, the supplementary holes are located in close proximity to the edge of the trumpet like tip. The model 3 is especially designed for usage in cavities (e.g., between the intestines in the abdominal cavity). For this purpose, the size of the suction head surface contacting the tissue is increased, and supplementary holes are evenly distributed at the surface. The bigger hollow cavity inside the suction head is filled with an open-pored polyurethane sponge to diminish turbulence in this area (cf. Table 1).

All three new models (1, 2, and 3) were tested using the same standardised in vitro setup, and the resulting flow-dependant noise emissions, noise maps, and the different amounts of secondary air intake were compared.

### 2.1. Statistics

Groups were compared by one-way ANOVA followed by Tukey's multiple comparisons tests. *p* values of less than 0.05 were defined as statistically significant. Statistical calculations were performed with GraphPad PRISM 7 (GraphPad Software Inc, La Jolla, US).

## 3. Results

**Figure 2:**
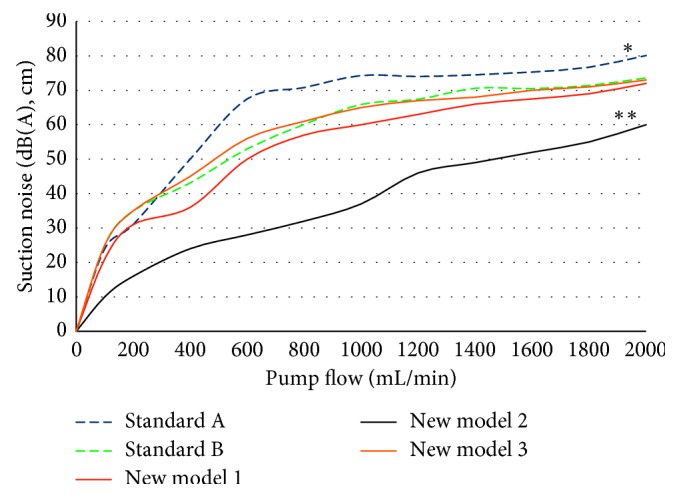
Sound pressure levels at different flow rates. An increase in the sound pressure level by 10 dB corresponds to a doubling of the perceived volume. ^*∗∗*^The new model 2 is highly significantly quieter at all pump rates over all other models (*p* < 0.001). ^*∗*^The standard A model is significantly louder against standard model B and new models 1 and 2 (*p* < 0.05).

Considerable differences were ascertained regarding the quantity and quality of emitted noises. As shown in Figure 2, the commercially available standard suction tips A and B emitted considerable noise with model A producing the highest noise levels due to its 22 side holes. For instance, model A produced 70 dB(A) (10 cm) even at low suction rates of 600 mL/min.

**Figure 3:**
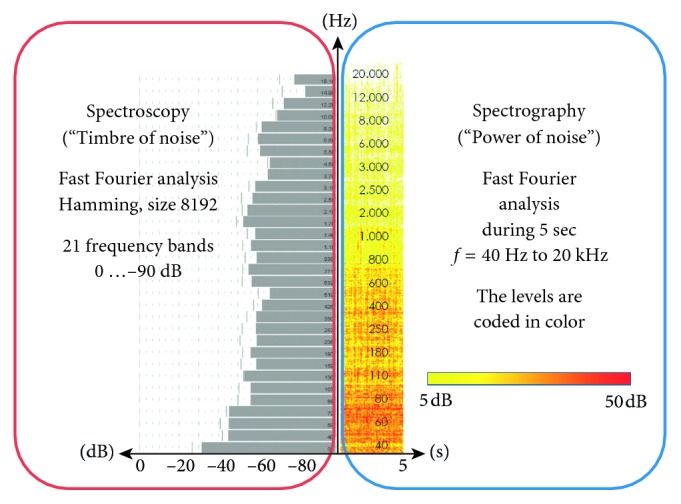
The spectroscopy corresponds to a snapshot of the frequency spectrum of the sound (“timbre” of noise). The spectrograph shows the noise over a longer period, and even slower frequency developments can be represented (“power” of noise).

**Figure 4:**
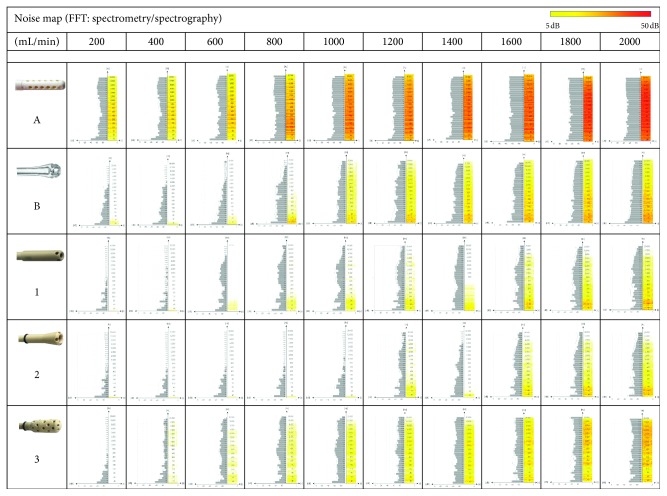
Noise maps for all five suction head models. The standard model A is noisy at low flow rates (full spectrum), the new models 1 and 2 show highly significant lower noise, especially at frequencies above 300 Hz, the standard model B is in lower flow rates significant quieter than the standard model A (Fast Fourier Analysis as spectroscopy and spectrography during 5 sec, Hamming-window, 20 Hz-20 kHz, FFT Size 8192, Sequoia 14.1.0.157 DC2, 64 bit, Magix Software GmbH, Berlin, Germany), noise map legend see Figure 3.

The frequency spectrum starts to show a level increase above 3 kHz (Figures 3 and 4). The optimized suction head tip, new model 2, is significantly quieter in all aspects. The frequencies above 3 kHz are significantly reduced (*p* < 0.001), and the overall improvement is apparent throughout the whole examined frequency range (Figures 2–4).

**Figure 5:**
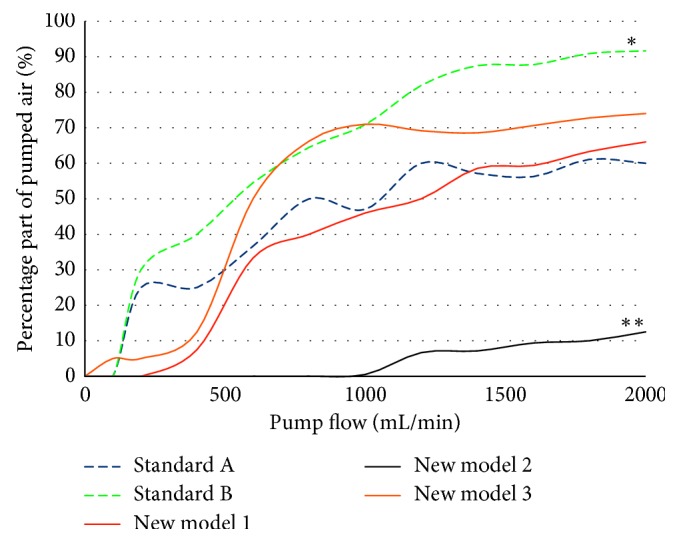
Percentages of pumped air at different flow rates. ^*∗∗*^ Compared to the other models, the new model 2 sucks air only at a pumping rate of more than 1000 mL/min (*p* < 0.001). ^*∗*^Model B vs. models A 1 and 3 is significant (*p* < 0.05).

Along with increasingly more audible noise emissions for increasing pump rates, the intake of secondary air also grows to significant portions (standard models A, B: flow at 1000 mL/min/air = 70%, at 2000 mL/min/air = 75–90%, Figure 5). The standard models A, B, and new model 1 behave significantly different compared to the new model 3 (*p* < 0.05), with the latter starting a massive intake of secondary air at flow rates of 500 ml/min. Only the new model 2 has shown a significantly reduction of secondary air even at higher suction rates (*p* < 0.001). The side holes that are oriented downward alter the inflow characteristics at the liquid-air boundary. Such modifications result in a significant later onset of flow disruptions (>1000 mL/min; Table 1; Figure 5).

## 4. Discussion

Conventional suction devices have a series of side holes to avoid tissue adhesion. However, these additional holes can cause air admixture during suctioning at the liquid-air boundary. Since parts of the additional holes are located above the liquid level, air is sucked in and leads to flow interruptions and considerable turbulences within the multiphase flow.

### 4.1. Physical Aspects

The interrupted flow is caused by immiscible blood and air with different viscosities (blood: *η* = 3–25 *µ*Pa·s, air: *η* = 17 *µ*Pa·s) [11]. The flow is also turbulent in most cases (such as the Reynolds number is more than 2500 in standard model A at a flow rate of 250 ml/min). Flow stoppages and turbulence lead to audible vibrations. The sound pollution of suctioning increases up to 120 dB(A) (100 cm) [4, 5, 7, 12], sound levels in suction devices peaked with smaller diameter (2 mm) between 4 and 6 kHz, with wider diameter (4 mm) around 3 kHz [13], although the diameter was positively correlated with sound energy [3], all perceived as noise. Noise is defined as “unwanted or undesirable sound” as well as “wrong sound in wrong place at wrong time” [14] for it may cause annoyance and decrease in work efficiency. In physics, it is regarded as random, fluctuating, inharmonious wave forms [15].

### 4.2. Aspects of Noise Pollution

The impact of noise on human performance depends on the type of noise and the task to be performed. Especially during critical periods and tasks, it may reduce mental efficiency and short-term memory [14]. Although there is a wide variability in individual sensitivity to noise [16], a normal healthy adult may tolerate about 50–55 dB(A) sound relatively well [17]. The World Health Organization (WHO) “Guidelines for community Noise” suggests that sound levels in hospital should not exceed 35 dB(A) L_Aeq_ [17]. Studies have shown that noise in the OT is even louder during critical components of the case and is related to equipment and staff, resulting in negative impact on patient safety [18]. It is said that the most important source of noise in the OT is the use of particular surgical tools [19]. Noise in health care settings has increased during the last 50 years [20].

At frequencies of 2.0–8.0 kHz (especially 3.0–4.5 kHz), the human ear has a higher sensitivity (the Fletcher–Munson curves of equal volume levels ISO 226: 2003), and sounds are perceived as being 10–20 dB louder than those outside this range, at same intensity [21]. Furthermore, in this frequency range, essential parts of speech information are located [22], impeding communication within the OT team. Persistent, high levels of noise are known to lead to health problems [23–26]. Noise is regarded as a general stressor [18] and a pervasive and influential source of stress [27], which may affect the cardiovascular system [28]. The volume level and the frequency of noise (sound quality) can have negative repercussions on the ability to concentrate [5, 6, 27], and it may represent a significant source of distraction [20, 29], although this is not unequivocal [30]. High levels of sound pollution may therefore influence outcome of surgical procedures [12, 31, 32] and provoke human errors [33]; inexperienced subjects are more prone to negative noise impact than experienced ones, particularly during difficult tasks [29]. Higher levels of noise were correlated directly with higher surgeons stress response (physiological and self-reported), as well as levels of surgical errors, putting patients at increased risks for postoperative complications [34], although the causal relation between noise and complications is hard to prove [35]. The US Agency for Healthcare Research and Quality mandates a “high-level priority” to reduce noise-induced distraction in the OT to improve patient safety, although, so far, little reliable and systematic information exists of the sound levels in the operating room environment [31]. Due to its inherently complex structure, errors can be catastrophic for patients and health care institutions alike [36]. Noise levels during operations have been correlated with surgical site infection (SSI) [37, 38], attributed to noise-induced distraction leading to lapses in compliance with aseptic principles.

As a result, it is advantageous for surgeons and patients to use a continuous quieter suction device. We were able to show that even small modifications in the geometry of suction heads make them significantly quieter.

Usually, sound pressure levels refer to a measuring distance of 1 m. The dimensions of the noise measuring stand (silent room) allow low-reflection measurements at a distance of 10 cm. In order to compare the SPL measured here with standard 1-m measurements, a correction must be made (minus 6 dB for every doubled distance). The closer the noise to the hearing organ itself is, the opposite effect is to be considered (increase of the sound pressure by 6 dB at half the distance). Suction noise near the inner ear (>100 dB(A), [3], especially in children during ear and neurosurgical procedures, has been described to result in lasting hearing loss [39]. However, tracheal suctioning in children (4–10 kHz, peak 96 dB) has not lead to measurable restrictions in hearing capacity/capability (24). In a prospective study, Nelson et al. [9] could not demonstrate lasting hearing loss due to ear canal suctioning, and Katzke et al. confirmed this finding [40, 41]. However, noise-induced hearing impairment may be more common than normally assumed [42], as the deterioration of hearing is hard to detect in the high-frequency range [43–45].

In our study, frequencies above the audible range (>16 kHz) were recorded (Figure 4). These high-frequency flow stoppages in particular are responsible for hemolysis and malactivation of leukocytes and platelets [46], although the exact mechanisms for the damage of blood cells are controversial [47–49]. The foaming [50, 51] or admixing of air (“aeration”) can adversely affect the integrity of the blood cells by direct oxygen contact. By reduction of air admixtures, membrane damage, oxidation of various blood components, and the formation of radicals can be avoided or reduced. Gentle and thus quiet suction would protect blood cells. The louder the suction noise, the greater the vibrations stress on the blood cells. Budde et al. have shown that avoiding turbulence (audible as a noise) reduces blood cell damage [52]. The technical solution for this is the Turbulence Controlled Suction System developed by Friedrich et al. [53]. Further studies have to show the impact of suction cup geometry in this relation.

### 4.3. Other Aspects

Air admixtures can also cause infection problems. In animal experiments, it has been shown that bacterial air contamination can be transported with the suction of secondary air [54]. That means that infectious complications may result from increased air mixing. In this respect, we present first improvements with our new model 2. However, the other modified models, 1 and 3 do not show significant changes in the proportion of extracted air. Nevertheless, the high-frequency vibrations are significantly reduced here as well. The new model 2 is very quiet (*p* < 0.001) and has shown a low level of aeration (*p* < 0.001).

Loudness in health care units disturbs communication, concentration, and increases stress. Engelmann et al. describe significant effects due to a noise reduction program in a pediatric operation theatre [8]. Through comparative measurements in the critical care environment, White and Zomorodi did show that there is a greater need for a viable solution [55]. Since not all noise sources can be controlled, Friedrich et al. developed the silent operating theatre optimisation system (SOTOS), a novel closed but flexible communication tool in noisy environment [56].

Our working group has shown that it is possible to reduce flow-induced noise and air admixture using a polypragmatic approach. The Turbulence Controlled Suction System (TCSS) adjusts the rotational speed of the roller pump system via a vibration sensor in the suction handle [53]. We can assume that the combination of TCSS and optimized suction head geometry should further reduce the noise level. The protective effect on integrity of blood cells has also been shown [52].

## 5. Conclusion

The flow-induced noise is correlated to the suction tip geometry. Parameters of the suction tip relevant to stream-flow can be improved. The optimized suction heads are significantly quieter, as shown in our experimental results. Such optimization may reduce noise-related hearing loss und reduce stress during surgery, as it leads to a more quiet operation theatre. A noise-optimized suction device can improve the performance of the surgical team, reduce complications, improve the quality of collected blood, reduce the need for allogenic transfusion and organ damage, and finally increase patient safety. Further studies and advanced techniques, such as computational fluid dynamics simulation, are necessary to continue the optimization on suction heads for various applications.

## Figures and Tables

**Table 1:** Suction tips of different models: standard models A and B are industrially manufactured disposable articles of daily use, and new models 1, 2, and 3 are our newly developed prototypes. Please notice, devices are patent protected (first line: photo, second line: CAD 3D model, and third line: technical drawing).

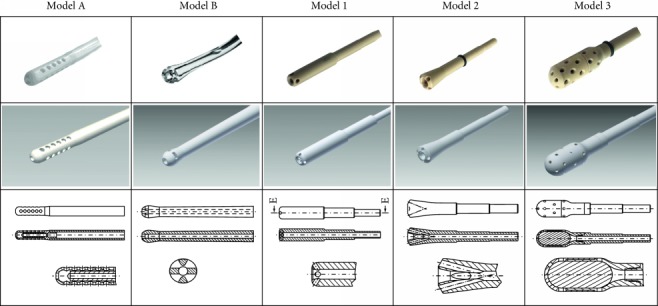

## Data Availability

The data used to support the findings of this study are available from the corresponding author upon request.
